# The Experience of Essential Tremor Caregivers: Burden and Its Correlates

**DOI:** 10.3389/fneur.2017.00396

**Published:** 2017-08-14

**Authors:** Sarah Morgan, Sarah Kellner, Jesus Gutierrez, Kathleen Collins, Brittany Rohl, Fanny Migliore, Stephanie Cosentino, Edward D. Huey, Elan D. Louis, Joan K. Monin

**Affiliations:** ^1^Department of Neurology, Division of Movement Disorders, Yale School of Medicine, Yale University, New Haven, CT, United States; ^2^Department of Neurology, College of Physicians and Surgeons, Columbia University, New York, NY, United States; ^3^Taub Institute for Research on Alzheimer’s Disease and the Aging Brain, College of Physicians and Surgeons, Columbia University, New York, NY, United States; ^4^Department of Psychiatry, College of Physicians and Surgeons, Columbia University, New York, NY, United States; ^5^Department of Chronic Disease Epidemiology, Yale School of Public Health, Yale University, New Haven, CT, United States; ^6^Center for Neuroepidemiology and Clinical Neurological Research, Yale School of Medicine, Yale University, New Haven, CT, United States; ^7^Department of Social and Behavioral Sciences, Yale School of Public Health, Yale University, New Haven, CT, United States

**Keywords:** clinical, essential tremor, caregiver burden, suffering, cognition

## Abstract

**Background:**

Essential tremor (ET) is associated with physical and cognitive impairments, as well as embarrassment, avoidance of social settings, and related difficulties that negatively impact the lives of patients. In similar disease contexts, burden on friends and relatives acting as caregivers has been noted and has well-documented implications. There has been no study examining caregiver burden related to ET.

**Methods:**

Data were gathered from 55 ET participants enrolled in a clinical study and their caregivers. The Zarit Burden Interview was used to assess caregiver burden. To assess clinical features that may be associated with burden, we collected several variables including the Montreal Cognitive Assessment, self-reported tremor disability, a videotaped neurological examination, questionnaires assessing ET participants’ suffering, caregivers’ perceptions of that suffering, and both caregiver and ET participant depressive symptoms. Spearman’s correlations were performed between caregiver burden and clinical features, and we created a multivariate linear regression model predicting caregiver burden.

**Results:**

Many ET caregivers provide little to no care and experience little to no burden. However, some caregivers (11%) provide over 25 h of care/week, and 13% experience high levels of burden. Caregivers most commonly provided assistance with writing and cooking. Increased burden was associated with the ET participants’ decreased cognition, more caregiving tasks, more hours/week of caregiving activities, a longer duration of care, more ET participant falls/year, more medications taken by the ET participant, and more depressive symptoms in both the ET participant and the caregiver (all *p* < 0.05). ET participants’ suffering and their caregivers’ perceptions of suffering were both associated with increased burden. Neither tremor severity score nor self-reported tremor disability score was associated with increased caregiver burden. Using a multivariate linear regression model, we found that caregivers’ increased perception of their partners’ suffering was the best predictor of caregiver burden.

**Conclusion:**

While not all relatives and friends of ET patients provide extensive care or experience high burden, there is a group reporting high levels of caregiver burden that requires the attention and counseling of clinicians. This burden is associated with primarily non-tremor symptoms of ET and with caregivers’ perception that their partners are suffering.

## Introduction

Essential tremor (ET) is a progressive neurological disease that can have a significant impact on patients’ activities and life satisfaction (1). Due to tremors, ET patients may experience functional disability (2) and diminished quality of life (3). Fine motor skills are particularly challenging; patients with ET often use modified utensils (4) or leave all writing tasks to a part of the day when tremors are mildest. Patients with more severe ET may be completely unable to use silverware or glassware without spilling all contents, and writing may be impossible (5). ET patients may also experience a host of additional non-tremor symptoms (6) including problems with balance (7–9), cognitive impairment compared with healthy controls (10–12), and sleep dysregulation (10, 13). Cognitive deficits are not uncommon, affecting 30–60% of patients (14), but run a wide range of severities from subclinical abnormalities to mild cognitive impairment (MCI) and dementia. Specific cognitive deficits in ET include impairments in executive function, working memory, verbal memory, and language (14, 15). Furthermore, individuals with ET also experience increased frailty as they age (16), and psychosocial factors contribute to the difficulty of dealing with ET. Many patients experience embarrassment (2, 17), anxiety (2, 17), and depression (8), with some patients meeting diagnostic criteria for social anxiety disorder and social phobia (18, 19).

Typically, the impairments due to ET are not severe enough to require constant care or paid caregivers. However, these challenges can require the assistance of relatives and friends who take on caregiving activities and act as caregivers. Patients may rely on family or friends to assist with eating, drinking, and dressing and to perform tasks such as writing checks or completing forms. In patients with voice tremors (approximated at 12% of all ET patients) (20), friends, and relatives will act as interpreters to assist the patient in being understood. Finally, ET caregivers provide emotional and psychological support. While we know that care providing can be burdensome across disease contexts, the experience of ET caregivers is unknown and is in need of exploration. In fact, a recent patient-needs assessment for ET identified “support for family members and caregivers” as an area of care that needed improvement (21).

In the literature, caregiver burden is defined as the diminishment of one’s emotional, physical, social, or financial well-being as a result of providing care (22). Caregiver burden has been well-described in patient populations that share similarities with ET, including the frail elderly (23), Parkinson’s disease (PD) (24), dementia (25), and MCI (26). In these studies, burden is associated with a pattern of patient and caregiver attributes. Typically, caregiver burden increases with disease severity and impaired patient cognition (16, 27). Additionally, large time investments by the caregiver have been found to be particularly burdensome, and both patient and caregiver mood seem to play an important role in moderating levels of caregiver burden.

This study has three aims: first, to assess the levels of caregiver burden in the ET population. Second, to determine the characteristics of both caregivers and ET patients that are correlated with burden. Based on previous work examining caregiver burden in similar populations, we hypothesize that increased tremor severity (14), longer duration of care (28), and cognitive impairment (29) will be associated with burden. Finally, previous research in multiple illness contexts has shown that caregivers who perceive their partner to be suffering experience a higher level of burden (23). Suffering as a construct is typically measured along three parameters (psychological, physical, and existential/spiritual distress) (30) and is assessed from the perspective of both the caregiver and the patient. Focusing on the dimensions of physical and psychological suffering, we will explore this relationship in the ET context. It is hypothesized that in ET as well, those caregivers who perceive more suffering experience more burden. This research aims to clarify the experiences of ET relatives and caregivers so that clinicians can provide the appropriate support and counseling.

## Materials and Methods

### Sample

The cohort of ET participants for these analyses of caregiver burden came from a larger longitudinal study of cognitive function in ET that began in July 2014 (Clinical Pathological Study of Cognitive Impairment in Essential Tremor, NINDS R01NS086736). The institutional review boards of Yale University and Columbia University approved that study, which aims to clinically and pathologically characterize a cohort of individuals with ET using motor, neuropsychiatric, and neuropsychological measures. Participants were recruited for this longitudinal study using advertisements on the International Essential Tremor Foundation webpage with the following eligibility criteria: (1) diagnosis of ET, (2) ≥55 years old, (3) willingness to perform study measures and be a brain donor, and (4) did not have deep brain stimulation surgery for ET. Data collection for research on caregiver burden began in October 2015 and ended in July 2016, and the current analyses considered the first 55 participants and their caregivers (designated by the participant as a person who could provide perspective on their well-being) who completed research assessments. Caregivers were recruited through conversation with each participant. Most caregivers (92.7%) were family members, primarily the children or spouses of participants. Assessment of clinical features, including cognition, tremor, and psychological factors, was conducted in participants’ homes by trained research assistants (Sarah Morgan, Sarah Kellner, Kathleen Collins, or Brittany Rohl). Data on caregivers’ experiences were collected *via* 30–60-min telephone interviews by trained study research assistants following the in-home visit.

### ET Participant Assessments

As a part of the larger longitudinal study, each participant took part in a 4–6 h clinical and cognitive assessment conducted by trained research assistants in participants’ homes around the United States. The assessment included a videotaped neurological examination and a series of questionnaires.

#### Demographics and Clinical Information

Data were collected on participants’ age, gender, disease history, number of falls in the past year, and medications (Table 1).

**Table 1:** Descriptive variables for ET participants (*n* = 55) and caregivers (*n* = 55).

ET participants		ET caregivers	
Age in years	76.9 ± 10.1 (56–97)	Age in years	66.6 ± 12.7 (41–89)
Female gender	35 (63.6%)	Female gender	35 (63.6%)
White race	53 (96.4%)	White race	50 (90.9%)
Education in years	16.5 ± 2.6	Education in years	16.3 ± 2.3
# of prescription medications	6.8 ± 10.8	Caregiver relationship to ET participant	
Total tremor score	23.1 ± 5.5	Spouse	31 (56.4%)
Tremor disability score	14.2 ± 4.9	Child	16 (29.1%)
Age at onset of tremor in years	41.4 ± 22.3 (5–78)	Friend	4 (7.3%)
Tremor duration in years	35.5 ± 21.5 (2–87)	Other (niece, daughter-in-law, girlfriend)	4 (7.3%)
Head tremor on examination	33 (60.0%)	Caregiver living with ET participant	34 (61.8%)
Voice tremor on examination	26 (47.3%)	Duration of care in years	6.7 ± 12.0 (0–63)
# of falls in past year	1.1 ± 2.5	Total assistance tasks	3.2 ± 3.0 (0–10)
GDS score	6.3 ± 5.5	Hours per week providing care	5.1 ± 9.9 (0–40)
MoCA score	24.8 ± 3.9 median = 26.0	CES-D-10 score	4.8 ± 4.8
CDR		Finds caregiving rewarding?	
0	40 (72.7%)	Very rewarding	26 (47.3%)
0.5	12 (21.8%)	Somewhat rewarding	19 (34.5%)
1	2 (3.6%)	Not at all rewarding	8 (14.5%)
2	1 (1.8%)	Unknown	2 (3.6%)
Physical suffering score	6.9 ± 3.4	Perceived physical suffering score	7.6 ± 4.9
Psychological suffering score	8.3 ± 7.5	Perceived psychological suffering score	10.7 ± 9.3
		ZBI-12 score	6.1 ± 8.2 (0–30)

*CDR, Clinical Dementia Rating; CES-D-10, Center for Epidemiological Studies Short Depression Scale; ET, essential tremor; GDS, Geriatric Depression Scale; MoCA, Montreal Cognitive Assessment; ZBI-12, Short Zarit Burden Inventory*.

#### Cognitive Ability

The Montreal Cognitive Assessment (MoCA) is a commonly used 30-point test of global cognition designed to detect MCI (31). Higher scores indicate higher cognitive abilities, and a score of 26 or lower is considered to be abnormal.

#### Depressive Symptoms

The Geriatric Depression Scale (GDS) is a validated, reliable 30-item inventory (range: 0–30) commonly used to assess depressive symptoms in the elderly (32) where higher scores indicate more depressive symptoms.

#### Physical and Psychological Suffering

Suffering was assessed by a reliable and valid scale with physical and psychological components (30). The seven-item physical suffering scale (range: 0–21) contains symptoms such as “fatigue,” “physical discomfort,” etc. The 18-item psychological suffering scale (range: 0–54) asks about both positive and negative feelings: “frustrated,” “cheerful,” “hopeless,” etc. We modified the wording of several items to best fit the ET population. Higher scores on both scales indicate more suffering. Both the physical and psychological scales show good internal consistency (Cronbach’s alphas = 0.66; 0.89). Participants self-administered both suffering scales and the GDS at the time of the visit with the assistance of study personnel.

#### Tremor Disability

A 10-item tremor disability questionnaire (range: 0–20, higher scores indicate greater disability) was administered (33, 34). Participants were asked about their difficulty with a range of daily activities: “carrying a cup of coffee,” “dialing a telephone,” etc. (0 = none, 1 = need to modify or loss of efficiency, 2 = disability). The questionnaire showed good validity, reliability, and internal consistency (Cronbach’s alpha = 0.96) (33).

#### Tremor Severity

The videotaped neurological examination was reviewed by a neurologist specializing in movement disorders (EDL) who used a reliable (35) and valid (36) clinical rating scale, the Washington Heights-Inwood Genetic Study of ET (WHIGET) tremor rating scale, to confirm ET diagnoses and to rate tremor severity. The valid and reliable (36) WHIGET diagnostic criteria required moderate or greater amplitude kinetic tremor during three or more tests or a head tremor, in the absence of PD, dystonia, or another cause (37). Severity scores were calculated from one postural tremor test and five kinetic tremor tests in each arm resulting in a total tremor score (12 total tests, range: 0–36) where higher scores indicated greater tremor (38). Head and voice tremor was noted as present or absent.

### Caregiver Interviews

Caregivers completed measures of their perceptions of ET participants’ cognitive and functional abilities, including a Clinical Dementia Rating (CDR) interview to evaluate functional abilities. The CDR ranges from 0 to 3 (0 = normal cognition, 0.5 = questionable dementia/MCI, 1 = mild dementia, 2 = moderate dementia, 3 = severe dementia) (39, 40). Caregivers were then asked to participate in a study concerning the experiences of relatives and caregivers of individuals with ET, and if amenable, they were verbally consented over the telephone as approved by the Yale University institutional review board. Data gathered during the telephone call included the following, which all pertained to the experiences of the caregivers themselves.

#### Demographics

Questionnaires also included inquiries into extent of care providing and assistance tasks (Table 1). Caregivers were asked to endorse the tasks with which they assisted participants from a list of 10 items (Table 2).

**Table 2:** Tasks of daily living with which ET caregivers provide assistance.

	Caregivers providing assistance, *n* (%)
Writing (e.g., signing name)	24 (43.6)
Cooking (e.g., chopping veggies, setting table)	18 (32.7)
Walking (e.g., losing balance)	17 (30.9)
Transportation (e.g., operating vehicle)	17 (30.9)
Eating (e.g., cutting food)	16 (29.1)
House/yard work (e.g., vacuuming, dishes, gardening)	16 (29.1)
Using a computer/phone (e.g., pressing correct buttons)	14 (25.5)
Drinking (e.g., pouring water, drinking from cup)	12 (21.8)
Dressing (e.g., buttons)	10 (18.2)
Administering medication (e.g., setting out pills)	7 (12.7)

*ET, essential tremor*.

#### Caregiver Burden

The Zarit Burden Inventory Short Form (ZBI-12) (range: 0–48) is a validated and reliable short form of the Zarit Burden Inventory (ZBI) used to measure burden experienced by caregivers of the elderly or disabled (41, 42). Caregivers endorse the frequency of each suggested feeling (e.g., “Do you feel angry around your relative?,” “Do you feel that your health has suffered because of your involvement with your relative?”) on a scale from 0 (never) to 4 (nearly always). Higher scores indicate more burden, and scores above 17 indicate high burden (43). The ZBI-12 showed good internal consistency: Cronbach’s alpha = 0.90 (Table 3).

**Table 3:** Caregivers’ responses to ZBI-12 and to ET-specific burden questions.

Do you feel…	Never (0)	Rarely (1)	Sometimes (2)	Quite frequently (3)	Nearly always (4)
**ZBI-12**					
…that because of the time you spend with your relative that you do not have enough time for yourself?	42 (76%)	4 (7%)	6 (11%)	3 (5%)	0 (0%)
…stressed between caring for your relative and trying to meet other responsibilities (work/family)?	39 (71%)	5 (9%)	7 (13%)	3 (5%)	1 (2%)
…angry when you are around your relative?	43 (78%)	5 (9%)	6 (11%)	1 (2%)	0 (0%)
…that your relative currently affects relationships with family members or friends in a negative way?	42 (76%)	5 (9%)	5 (9%)	2 (4%)	1 (2%)
…strained when you are around your relative?	38 (69%)	6 (11%)	4 (7%)	5 (9%)	2 (4%)
…that your health has suffered because of your involvement with your relative?	51 (92%)	1 (2%)	1 (2%)	1 (2%)	1 (2%)
…that you do not have as much privacy as you would like because of your relative?	49 (89%)	1 (2%)	2 (4%)	2 (4%)	1 (2%)
…that your social life has suffered because you are caring for your relative?	46 (84%)	2 (4%)	2 (4%)	4 (7%)	1 (2%)
…that you have lost control of your life since your relative’s illness?	51 (93%)	2 (4%)	0 (0%)	1 (2%)	1 (2%)
…uncertain what to do about your relative?	30 (55%)	7 (13%)	9 (16%)	5 (9%)	4 (7%)
…you should be doing more for your relative?	32 (58%)	8 (15%)	7 (13%)	6 (11%)	2 (4%)
…you could do a better job in caring for your relative?	35 (64%)	7 (13%)	8 (15%)	3 (5%)	2 (4%)
**ET-specific burden questions**					
…worried about the assumptions others might make about your relative due to their tremor?	48 (87%)	2 (4%)	3 (5%)	1 (2%)	1 (2%)
…embarrassed that you need to assist your relative with daily activities?	54 (98%)	1 (2%)	0 (0%)	0 (0%)	0 (0%)
…concerned about how your relative’s illness will progress over time?	13 (24%)	11 (20%)	12 (22%)	11 (20%)	8 (15%)
…concerned that you assist your relative more than they actually need?	48 (87%)	2 (4%)	1 (2%)	2 (4%)	2 (4%)
…concerned that your assistance prevents your relative from achieving his/her maximum potential independence?	47 (85%)	2 (4%)	2 (4%)	1 (2%)	3 (5%)

*ET, essential tremor; ZBI-12, Short Zarit Burden Inventory*.

#### ET-Specific Caregiver Burden

To quantify ET-specific caregiver burden, five Likert-scale questions with options 0 (never) to 4 (always) were asked (Table 3). We summed these responses to create an ET-specific caregiver burden score with a range of 0–20, where higher scores indicate more ET-specific burden.

#### Depressive Symptoms

The Center for Epidemiological Studies Depression Scale (CES-D) is a validated measure for assessing depression in the general population (44). The 10-item version (CES-D-10, range: 0–30, with higher scores indicating more depressive symptoms) includes statements such as “I felt hopeful about the future” and “I felt lonely” and has been shown to be reliable and valid (45).

#### Perceived Suffering of ET Participant

The perceived suffering scales mirror the ET participants’ suffering scales (see above) but asked the caregiver their perspective on how often the participant experienced each item. The physical and psychological measures are reliable and valid and both showed good internal consistency (Cronbach’s alphas = 0.73; 0.89) (30).

### Statistical Analyses

Statistical analyses were performed using SPSS software (version 21.0; IBM Corps). To describe demographics (Table 1) and the specific tasks with which caregivers provided assistance (Table 2), mean and SDs as well as percentages are presented. Percentages are also presented to describe burden experienced by ET caregivers (Table 3). The ZBI-12 scores were not normally distributed, so to examine ET participant and caregiver characteristic correlates of caregiver burden, non-parametric tests (Spearman’s rank correlations and Mann–Whitney *U* tests) were used (Table 4).

**Table 4:** Spearman’s correlations with ZBI-12 score.

ET participant	*r_s_*	*p*-value	ET caregiver	*r_s_*	*p*-value
Age	0.17	0.21	Age	−0.21	0.12
# of prescription medications	0.34	0.01	Duration of care (in years)	0.39	0.00
# of falls in past year	0.32	0.02	Total assistance tasks	0.37	0.01
Total tremor score	0.13	0.33	Hours/week providing care	0.53	0.00
Tremor disability score	0.05	0.71	CES-D-10 score	0.32	0.02
Tremor duration (in years)	0.18	0.18	Perceived physical suffering score	0.41	0.00
GDS score	0.30	0.03	Perceived psychological suffering score	0.61	0.00
CDR	0.51	0.00			
MoCA score	−0.47	0.00			
Physical suffering score	0.09	0.51			
Psychological suffering score	0.28	0.04			

*r_s_, Spearman’s rho; CES-D-10, Center for Epidemiological Studies Short Depression Scale; CDR, Clinical Dementia Rating; ET, essential tremor; MoCA, Montreal Cognitive Assessment; ZBI-12, Short Zarit Burden Inventory; GDS, Geriatric Depression Scale*.

To examine the hypothesis that perceptions of suffering would be associated with burden, a linear regression model was performed that assessed the ability of caregivers’ perceptions of physical and psychological suffering to predict caregiver burden above and beyond other patient and caregiver characteristics (Table 5). Our model included covariates that were associated with both the dependent variable (ZBI-12 score) and the independent variables (perceived physical and psychological suffering scores) at the *p* < 0.05 level during initial correlation analyses. These variables were: number of prescription medications, duration of care providing, total assistance tasks, hours/week providing care, GDS score, CDR, MoCA score, and psychological suffering score (as reported by ET participant). ZBI-12 scores were not normally distributed. To meet model assumptions of normality, ZBI-12 scores underwent square-root transformation. The model met the assumptions of linearity, homoscedasticity, independence, and normality.

**Table 5:** Linear regression model predicting ZBI-12.

	β coefficient	*p*-Value
# of prescription medications	−0.05	0.65
Duration of care (in years)	0.16	0.14
Total assistance tasks	0.01	0.92
Hours/week spent providing care	−0.06	0.70
GDS score	−0.25	0.19
CDR	0.15	0.39
MoCA score	−0.10	0.52
Psychological suffering score (reported by participant)	0.09	0.67
Perceived psychological suffering score (perceived by caregiver)	0.61	0.00
Physical suffering score (perceived by caregiver)	0.26	0.06

*CDR, Clinical Dementia Rating; GDS, Geriatric Depression Scale; MoCA, Montreal Cognitive Assessment; ZBI-12, Short Zarit Burden Inventory*.

## Results

Fifty-five pairs of ET participants and their caregivers completed all necessary questionnaires for analysis. ET participants had a mean age of 76.9 years and a mean total tremor score of 23.1 (Table 1). Most caregivers were the spouses (31, 56.4%) or children (16, 29.1%) of ET participants. Four caregivers (7.3%) were friends, and four (7.3%) had another familial relationship (long-term girlfriend, daughter-in-law, niece) with the ET participant. Caregivers were on average younger than ET participants with a mean age of 66.6 (*t* = −5.76, *p* < 0.01) (Table 1).

Caregivers perceived higher psychological suffering scores than ET participants reported (*t* = 2.53, *p* < 0.05). Caregivers perceived similar physical suffering scores to what ET participants reported (*t* = 1.06, *p* = 0.29) (Table 1).

### Aim 1: Quantifying ET Caregiver Assistance and Caregiver Burden

Caregivers assisted with an average of 3.2 tasks (out of 10) and had been providing an average of 5.1 h of care per week for 6.7 years (Table 1). However, within those averages, caregiving varied from very low to very high. Task assistance ranged from 0 tasks to all 10 of the items on the survey. Seventeen caregivers (31%) reported providing no care, yet 6 caregivers (11%) reported over 25 and up to 40 h/week of care providing, and 11 (20%) reported a duration of 10 or more years.

The most common task requiring caregiver assistance was writing, with 24 caregivers (43.6%) providing help. All except two caregiving tasks (administering medication and dressing) were reported by over 20% of caregivers (Table 2).

Caregivers reported a mean ZBI-12 score of 6.1 ± 8.2 (Table 1). Fifteen caregivers (27%) reported no burden, but 7 caregivers (12.7%) reported levels of burden above 17, indicating high burden (43).

Within individual ZBI-12 items, caregivers most frequently endorsed feeling “strained when around (their) relative,” “uncertain about what to do with (their) relative,” and that they “should be doing more for (their) relative,” with 7 (13%), 9 (16%), and 8 (15%) caregivers, respectively, reporting these feelings “quite frequently” or “nearly always” (Table 3). In answering the ET-specific burden questions, 42 (76%) caregivers reported “feeling concern about how (their) loved one’s tremor will progress over time,” and 19 (34.5%) caregivers reported that feeling “quite frequently” or “nearly always” (Table 3).

### Aim 2: Correlates of Caregiver Burden

Bivariate analyses showed participants’ decreased cognition (lower MoCA score, higher CDR) and increased depressive symptoms (higher GDS score) were related to greater caregiver burden. Additionally, more prescription medications and more falls in the past year were associated with greater caregiver burden (Table 4). Finally, there was an association between caregiver burden and participants’ psychological suffering score (*r* = 0.28, *p* < 0.05). Burden was not correlated with tremor severity score or tremor disability score. Neither older age of ET participants nor longer tremor duration was associated with caregiver burden (Table 4). A Mann–Whitney *U* test found no significant difference in burden between the caregivers of male or female participants.

For caregivers, more burden was related to more care: higher ZBI-12 scores were positively associated with increased assistance tasks, more hours/week providing care, and longer duration of care providing. Burden (measured by ZBI-12 scores) was also strongly associated with perceived physical and psychological suffering scores (*r* = 0.41, *p* < 0.01; *r* = −0.61, *p* < 0.01, respectively). Finally, caregiver depressive symptoms were also related to burden: ZBI-12 scores were associated with increased CES-D-10 scores (Table 4). Mann–Whitney *U* tests found no significant difference in burden between caregivers who were: male/female, spouse/child, cohabiting/non-cohabitating, or between those who found caregiving rewarding/not rewarding.

### Aim 3: Suffering As a Predictor of Burden

The linear regression model predicting square root-transformed ZBI-12 score included perceived physical and psychological suffering scores as well as ET participant characteristics (number of prescription medications, GDS score, CDR, MoCA score, and psychological suffering score as reported by participant) and measures describing caregiving (duration of care providing, total assistance tasks, and hours/week providing care). The final model predicted 55.0% (adjusted *R* square) of variance in ZBI-12 score, and perceived psychological suffering score was the only independent predictor of burden (*p* = 0.00) with a trend seen for perceived physical suffering (*p* = 0.06, Table 5).

### Further Analyses

To address the idea that the co-presence of dementia may be driving effects on burden, we conducted further analysis. When only non-demented participants were included (CDR < 1, *n* = 52), overall results changed minimally. The average ZBI-12 score became 5.2 ± 7.8 (range: 0−29), and six caregivers (11.5%) reported high levels of burden—when compared with seven caregivers (12.7%) who reported high levels of burden in the total group of both demented and non-demented participants. Caregivers of non-demented participants provided an average of 4.0 h of care per week (versus 5.1 h in the larger sample), and four caregivers (7.8%) provided over 25 h per week [versus six caregivers (10.9%) in the larger sample]. In bivariate analyses, lower age of caregivers became significantly correlated with burden (*r* = −0.29, *p* < 0.05) whereas it had not been associated in the larger sample, and psychological suffering score as reported by the ET participant was no longer associated with burden. All other associations with ZBI-12 score that were significant in the original population remained significant, including correlations between participants’ cognitive functioning (CDR and MoCA score) and ZBI-12. After recreating the linear regression model with the exclusion of demented participants, perceived psychological suffering score remained the best predictor of burden (β = 0.64, *p* = 0.003).

To further assess whether caregiver burden might also be present in our sample even after having removed participants with even milder forms of cognitive impairment, we repeated our analyses excluding any participants with either dementia or MCI (i.e., the 40 included participants all had CDR = 0) and again found that results changed minimally. The average ZBI-12 score became 4.3 ± 7.5 (range: 0−29), and five caregivers (12.5%) reported high levels of burden. The percentage of caregivers experiencing high levels of burden remained very consistent throughout analyses (among all 55 participants = 12.7%, among 52 non-demented participants = 11.5%, and among 40 cognitively normal participants = 12.5%). Caregivers of cognitively normal ET participants provided an average of 3.0 h of care per week, and two (5.0%) provided over 25 h of care per week. In bivariate analyses, correlation coefficients were similar, and the associations between ZBI-12 score and duration of care, hours/week of care, BDI-10 score, and perceived psychological suffering remained significant. No variables were significantly associated with ZBI-12 score in the cognitively normal group that had not previously been associated in the full sample. In a linear regression model including only the 40 cognitively normal ET participants, perceived psychological suffering again remained the best predictor of burden (β = 0.48, *p* = 0.024).

## Discussion

Caregiver burden has not been previously assessed in the context of ET. This study sought to understand the level of caregiver burden in ET, to discover the clinical correlates of such burden, and to investigate the relationship between perceived patient suffering and burden.

We found that while some relatives and loved-ones of our ET participants are providing little care, others are very active caregivers. There are ET caregivers who spend a significant amount of time (up to 40 h/week) providing care and assisting their partners with 10 (out of 10) activities of daily living. On average, caregivers are helping with three of these daily living tasks, and almost half (43.6%) of all of the caregivers surveyed are assisting with writing tasks. We found as well that this caregiving can be burdensome. Our caregivers reported a mean ZBI-12 score of 6.1 ± 8.2. Other studies using the same scale to assess caregiver burden found means of 15.0–17.7 for dementia patients (46, 47), 9.6 for heart failure patients (48), 11.1–12.0 for advanced cancer patients (46, 48), and 21.7 for patients with acquired brain injury (46). Average ET ZBI-12 scores are slightly lower than in these other studied conditions. Yet, the range of ZBI-12 scores that we found in the ET population is close to matching these other conditions. The 10th–90th percentile range of ZBI-12 scores for our ET participants was 0–20.2. For heart failure patient caregivers, the 10th–90th percentile range was 0–22 (48), and for advanced cancer patient caregivers, the range was 0–24.2 (48). 12.7% of our ET caregivers reported levels of burden within the highest quartile level found by the creators of the ZBI-12 (43). This compares to findings of 30 and 19% in the lung cancer and heart failure caregiver population, respectively. While many ET caregivers do not experience much burden, we have found that there is a group who are burdened at levels that are within the range of other difficult and disabling conditions.

To our knowledge, there have been no previous studies of caregiver burden in ET, so we cannot compare our results with earlier findings. However, the patient and caregiver characteristics that correlated with burden in this study are largely in-line with research on PD caregiver burden or MCI caregiver burden. Like past studies, we found burden to be unrelated to caregivers’ age but significantly related to the amount of time and number of tasks that caregivers provided assistance (24). Predictably, years spent providing care, hours per week dedicated to caregiving, and the number of tasks with which caregivers assisted were all associated with increased reports of burden. Also in-line with previous research in the PD and MCI populations, we found that ET participants’ decreased cognition was significantly associated with increased burden for their caregivers (29). Functional cognition (assessed by the CDR) and global cognition (assessed by the MoCA) were both related to caregiver burden, and these relationships remained intact in a group of participants with normal cognition or only MCI (as defined by CDR < 1). Although one might assume tremor severity to be an important indicator of burden, we did not find associations between burden and either objective tremor severity scores (as measured by a neurologist) or subjective tremor disability scores (as reported by the ET participants). In past PD research, some studies have found caregiver burden to be connected to tremor severity and some have not (24, 49). Our results point toward the non-tremor symptoms of ET being more burdensome to caregivers than the tremor symptoms.

Our findings also suggest a relationship between depression and caregiver burden that likely warrants further exploration in the context of ET. Like past studies in other populations, we found that increased depressive symptoms in either the ET participant or in the caregiver were associated with higher burden for caregivers (24). Perhaps it is particularly burdensome for caregivers to witness their loved one experience depressive symptoms.

Similarly, we found caregiver perceptions of suffering to be strongly associated with caregiver burden whereas measures of patient impairment (total tremor score, tremor disability score) were not. These findings fit past research which describes perceived suffering as a significant predictor of caregiver burden even after adjusting for disability (50). Our data support the hypothesis that dealing with a partner’s suffering (especially psychological suffering) is difficult for caregivers and can outweigh the burden of actual tasks or tangible assistance that caregivers also provide.

This study is limited in that we did not include a group of healthy, age-matched controls, or a similar disease population. As no studies of PD or MCI have used the ZBI-12 to assess caregiver burden, we cannot compare directly to either population. A control group would have helped us to place results in an age-matched context to confirm that the results are disease specific and not merely aging related. A second limitation is that our sample came from a population of ET cases who had agreed to become brain donors, which could have biased our sample toward people with more severe tremors. However, in our analyses, we found no correlation between total tremor score and caregiver burden (*r* = 0.13, *p* = 0.33, Table 4) or between tremor disability score and caregiver burden (*r* = 0.05, *p* = 0.71, Table 4). This lack of association suggests that the potential skewing of our sample toward more severe tremor is unlikely to have affected our main results. Furthermore, our sample was not exclusively comprised of ET cases with severe tremor; 11 participants (20.0%) were assigned WHIGET tremor ratings of 1 (low amplitude) or 1.5 (only occasionally moderately amplitude) on all items of the videotaped neurological examination (36). An additional limitation of the study is that the ET cases were of advanced age and on average had the tremor for more than 30 years. Studies of ET populations with different characteristics (e.g., younger age or shorter duration) would be of value and would likely yield different results. Strengths of this study are our participants’ wide range of tremor severity and cognitive abilities and our inclusive definition of the term “caregiver.” We were able to broadly assess the burden on the relatives and friends of ET patients.

Why is caregiver burden important to consider? Research has shown that caregivers struggling with burden can be immensely impacted by their responsibilities. Burden has been repeatedly linked with a lower quality of life (51) and depression (49). Given our results, we can identify a simple and likely effective way of reducing burden. We found that perceived suffering was an important predictor of burden and also that caregivers over-reported suffering when compared with ET participants. Conversations in which caregivers are brought to understand the actual magnitude of suffering felt by ET patients would decrease perceived suffering and thus begin to reduce burden.

In conclusion, we were able to answer the three aims that this research set out to explore. We found that some caregivers of ET patients experience moderate to high levels of burden and that burden is associated with decreased cognition, the level of care providing, and depressive symptoms in patients and caregivers—but is not associated with tremor severity. Caregivers’ perception that their partners were psychologically suffering was also associated with burden and in fact, was the best predictor of caregiver burden. While not all relatives and friends of ET patients provide extensive care or experience burden, there is a group reporting high levels of caregiver burden that requires the attention and counseling of clinicians. This burden is associated with primarily non-tremor symptoms of ET and with caregivers’ perception that their partners are suffering.

## Ethics Statement

The cohort of ET participants for these analyses of caregiver burden came from a larger longitudinal study of cognitive function in ET that began in July 2014 (Clinical Pathological Study of Cognitive Impairment in Essential Tremor, NINDS R01NS086736). The institutional review boards of Yale University and Columbia University approved that study, which aims to clinically and pathologically characterize a cohort of individuals with ET using motor, neuropsychiatric, and neuropsychological measures.

## Author Contributions

SM was involved in the conception and design of this work, the analysis and interpretation of data, the drafting of the manuscript, and she gives final approval of this version to be published and agreement to be accountable for all aspects of the work in question. SK was involved in the conception and design of this work, the acquisition of data, the critical revision of the manuscript, and she gives final approval of the version to be published and agreement to be accountable for all aspects of the work in question. JG was involved in the conception and design of this work, the acquisition of data, the critical revision of the manuscript, and he gives final approval of the version to be published and agreement to be accountable for all aspects of the work in question. KC was involved in the conception and design of this work, the acquisition of data, the critical revision of the manuscript, and she gives final approval of the version to be published and agreement to be accountable for all aspects of the work in question. BR was involved in the conception and design of this work, the analysis and interpretation of data, the critical revision of the manuscript, and she gives final approval of the version to be published and agreement to be accountable for all aspects of the work in question. FM was involved in the conception and design of this work, the analysis and interpretation of data, the critical revision of the manuscript, and he gives final approval of the version to be published and agreement to be accountable for all aspects of the work in question. SC was involved in the conception and design of this work, the analysis and interpretation of data, the critical revision of the manuscript, and he gives final approval of the version to be published and agreement to be accountable for all aspects of the work in question. EH was involved in the conception and design of this work, the acquisition of data, the critical revision of the manuscript, and he gives final approval of the version to be published and agreement to be accountable for all aspects of the work in question. EL was involved in the conception and design of this work, the acquisition of data, the critical revision of the manuscript, and he gives final approval of the version to be published and agreement to be accountable for all aspects of the work in question. JM was involved in the conception and design of this work, the analysis and interpretation of data, the drafting of the manuscript, and she gives final approval of this version to be published and agreement to be accountable for all aspects of the work in question.

## Conflict of Interest Statement

The authors declare that the research was conducted in the absence of any commercial or financial relationships that could be construed as a potential conflict of interest.
